# Next-generation sequencing in dermatology

**DOI:** 10.3389/fmed.2023.1218404

**Published:** 2023-09-29

**Authors:** Andrew D. King, Hany Deirawan, Paytra A. Klein, Bahar Dasgeb, Catherine I. Dumur, Darius R. Mehregan

**Affiliations:** ^1^Department of Dermatology, Wayne State University School of Medicine, Detroit, MI, United States; ^2^Albany Medical College, Albany, NY, United States; ^3^Department of Surgical Oncology, Rutgers Cancer Institute of New Jersey, New Brunswick, NJ, United States; ^4^Bernhardt Laboratories, Sonic Healthcare Anatomic Pathology Division, Jacksonville, FL, United States

**Keywords:** next-generation sequencing, skin cancer, melanoma, squamous cell carcinoma, cutaneous lymphoma, genodermatoses

## Abstract

Over the past decade, Next-Generation Sequencing (NGS) has advanced our understanding, diagnosis, and management of several areas within dermatology. NGS has emerged as a powerful tool for diagnosing genetic diseases of the skin, improving upon traditional PCR-based techniques limited by significant genetic heterogeneity associated with these disorders. Epidermolysis bullosa and ichthyosis are two of the most extensively studied genetic diseases of the skin, with a well-characterized spectrum of genetic changes occurring in these conditions. NGS has also played a critical role in expanding the mutational landscape of cutaneous squamous cell carcinoma, enhancing our understanding of its molecular pathogenesis. Similarly, genetic testing has greatly benefited melanoma diagnosis and treatment, primarily due to the high prevalence of BRAF hot spot mutations and other well-characterized genetic alterations. Additionally, NGS provides a valuable tool for measuring tumor mutational burden, which can aid in management of melanoma. Lastly, NGS demonstrates promise in improving the sensitivity of diagnosing cutaneous T-cell lymphoma. This article provides a comprehensive summary of NGS applications in the diagnosis and management of genodermatoses, cutaneous squamous cell carcinoma, melanoma, and cutaneous T-cell lymphoma, highlighting the impact of NGS on the field of dermatology.

## Introduction

Next,-generation sequencing (NGS) is a high-throughput nucleotide sequencing method that allows simultaneous sequencing of massive amounts of DNA reads in parallel. Since its introduction, NGS has revolutionized the field of genomics as it allows for fast and scalable sequencing of human genomes at a lower cost. The technical capabilities allowed by NGS heralds improvement in clinical diagnostics and is especially exciting in the field of dermatology.

The chain termination method of determining the sequence of nucleotides in a DNA fragment, developed in 1977, was the first DNA sequencing method. Sanger sequencing has over 99.9% accuracy and is considered the gold standard for nucleic acid sequencing (1). However, this method is time-consuming and expensive as it can only sequence small genomic regions (approximately 300 to 1,000 base pairs) at a time (2). This costs approximately $500 USD per megabase amounting to $1.5 million to sequence an entire human genome. These time and cost limitations sparked an increased demand for novel DNA sequencing methods that were faster and cheaper, which led to the advent of NGS. NGS can sequence an entire human genome for less than $0.50 per megabase (3). Ongoing efforts to drive down the cost have achieved costs as low as $1 per gigabase, resulting in genomes costing $100 to sequence (4). This reduction in time and cost has led to increased access to sequencing technologies and a subsequent explosion in research and clinical diagnostics (5).

With regards to DNA sequencing, techniques can be broadly categorized into three main scales: whole-genome sequencing (WGS), whole-exome sequencing (WES), and targeted sequencing. WGS spans the entire genome and can detect mutations in both protein-coding and non-protein-coding DNA regions (6). WES restricts sequencing to the exome, which targets protein-coding regions, splice junctions, neighboring gene regulatory regions (e.g., promoter, untranslated regions), as well as non-coding RNAs. Exomes comprise approximately 1 % of the genome (5). Since exonic mutations represent most known disease-causing mutations, WES is considered a cost-effective and preferred alternative to WGS (5, 7). Lastly, targeted sequencing involves limiting sequencing to specifically selected sets of genes or genomic regions and is thus cheaper and more commonly used when a specific disease is suspected.

NGS platforms mostly share similar steps, starting with generation of nucleic acid libraries that consist of small fragments of DNA with ligated adapters. The libraries are then amplified and bound to a substrate (e.g., patterned flow cells that contain billions of nanowells), where individual unique DNA fragments form clusters. In the most common method, sequencing by synthesis, the nucleotide sequence is detected through visualization of fluorescence brought on by the addition of a modified nucleotide to a growing DNA strand. This is cyclically repeated for the length of the short DNA fragments spanning 50 to 150 base pairs. Digital sequences collected from sequencing devices are then sent through quality control and mapped to a reference genome. Further analysis can be performed and varies based on the specific application.

In this review, we will review the application of NGS in different areas of dermatology with a focus on clinical studies in genodermatoses, melanoma, keratinocyte carcinoma, and cutaneous lymphoma. Other applications such as use in inflammatory conditions, infectious diseases, and microbiome studies are not included in the scope of this review. Similarly, our discussion is limited to DNA sequencing.

## Genodermatoses

The ability of next generation sequencing to resequence the human genome at a massive scale has made a tremendous impact in the area of genetic skin diseases, both in terms of discovery and diagnosis. There has been a plethora of discoveries made with NGS, including identification of underlying somatic mosaic mutations in *IDH1* and *IDH2* in Maffucci syndrome and germline *CHST8* mutations in autosomal recessive peeling skin syndrome (8, 9). NGS has also been used to identify novel genes in genodermatoses with well-established causes, such as the discovery of novel germline *EXPH5* mutations in epidermolysis bullosa (EB) (10). The topic of discovery has been recently reviewed by Chiu and colleagues who show 166 new disease-gene associations, 35 of which were novel uncharacterized diseases between 2009 and 2019 since NGS technology has entered clinical use (11).

The utility of NGS in dermatology clinics for use in diagnosis of genodermatoses has not yet reached its full potential due to limited understanding of the complete genetic basis of hundreds of diverse disorders. One key challenge in the use of NGS in clinical diagnosis is its poor diagnostic yield. Of the first 250 patients referred for whole exome sequencing in a single center in 2011, only 25% yielded a molecular diagnosis (12). This was considered higher than other genetic tests, such as karyotype (5-15%), chromosomal microarray (15-20%), and Sanger sequencing. A recent meta-analysis reported mean rate of diagnosis among 37 studies of genetic disease to be 31% (13).

NGS is increasingly being utilized in dermatology clinics for diagnosing classic Mendelian genodermatoses with well-defined genetic underpinnings, particularly in diseases with skin fragility and disorders of cornification exemplified by epidermolysis bullosa and ichthyosis, respectively. These two categories will be the focus of this section.

### Disorders with skin fragility – epidermolysis bullosa

Disorders with skin fragility are a group of genetic skin conditions characterized by peeling or blistering of the skin due to decreased mechanical resilience. Epidermolysis bullosa is the prototypical disease of this group, which is itself a heterogeneous disease divided into four main types and over 30 clinical subtypes. Underlying this disease are up to 21 different genes and up to 47 for the broader group of disorders with skin fragility (14, 15).

Traditionally, the diagnosis of EB has relied on the identification of candidate genes with immunofluorescence antigen mapping (IFM) and transmission electron microscopy (TEM), followed by confirmation using Sanger sequencing. Given the large number of possible genes and difficulty distinguishing between clinical subtypes early in the disease course, diagnosis has required these former steps prior to identification of genetic mutations. However, NGS is an ideal tool enabling parallel sequencing of many genes, which is not feasible with Sanger sequencing. For example, the sequencing of *COL7A1* alone requires more than 70 primer pairs to cover its 118 exons and flaking introns using Sanger sequencing (16). Guidelines for diagnosis of EB have already incorporated NGS in molecular testing of EB (17, 18). Furthermore, retrospective analysis of EB patients in North America has shown increasing use of genetic testing over the past 30 years, with the highest rates since the introduction of NGS (19).

Several studies over the past decade have explored use of NGS in the clinical diagnosis of EB (Table 1). Outside of individual case reports used for discovery of gene mutations, clinical NGS with both WES and targeted sequencing panels were used for diagnosis of EB patients starting in 2015. In one study from the United Kingdom, WES was able to identify pathogenic mutations in all 9 patients for whom biopsy analysis and Sanger sequencing failed (16). Similarly, a group from Italy developed a 34 gene targeted sequencing panel that successfully identified predominately pathogenic germline mutations in all 8 trios with previously unknown genetic diagnoses (27). Of note, the targeted sequencing pipeline allowed identification of mutations in a 72-h procedure by utilizing the low throughput Ion PGM platform.

**Table 1:** Epidermolysis bullosa cohorts with clinical NGS testing.

# of patients	Yield	Platform	Panel size (genes)	Region	Reference
138	100%	Targeted	19 *COL17A1, COL7A1, DSP, DST, EXPH5, FERMT1, ITGA3, ITGA6, ITGB4, KLHL24, KRT14, KRT5, LAMA3, LAMB3, LAMC2, PKP1, PLEC, TGM5*	China	(20)
91	84%	Targeted	21 *CD151, CDSN, CHST8, COL17A1, COL7A1, DSP, DST, EXPH5, FERMT1, ITGA3, ITGA6, ITGB4, JUP, KRT14, KRT5, LAMA3, LAMB3, LAMC2, PKP1, PLEC, TGM5*	Iran	(21)
87	94%	Targeted	11 *COL7A1, COL17A1, FERMT1, ITGB4, KRT14, KRT5, LAMA3, LAMB3, LAMC2, PLEC, TGM5*	Brazil	(22)
57	100%	WES	--	China	(23)
43	98%	Targeted	21 *CD151, CDSN, CHST8, COL7A1, COL17A1, DSP, DST, EXPH5, FERMT1, ITGA3, ITGA6, ITGB4, JUP, KRT14, KRT5, LAMA3, LAMB3, LAMC2, PKP1, PLEC1, TGM5*	United States	(24)
40	90%	Targeted	49 *ARHGAP31, CD151, CDSN, CHST8, COL16A1, COL17A1, COL23A1, COL7A1, CSTA, CTGF, DCN, DSC3, DSG1, DSG2, DSG3, DSG4, DSP, DST, EXPH5, FERMT1, FLII, GRIP1, ILK, ITGA2, ITGA3, ITGA5, ITGA6, ITGB1, ITGB4, JUP, KLHL24, KRT14, KRT5, KRT6A, KRT6C, LAMA3, LAMA5, LAMB1, LAMB3, LAMC1, LAMC2, MMP1, NID1, NID2, PKP1, PLEC, SOX18, SOX7, TGM5*	Germany	(25)
21	95%	WES	--	India	(26)
9	100%	WES	--	United Kingdom	(16)
8	100%	Targeted	34 *ATP2A2, CD151, COL17A1, COL1A1, COL7A1, CSTA, DSP, EXPH5, FERMT1, FREM1, GRIP1, ITGA2, ITGA2B, ITGA3, ITGA5, ITGA6, ITGB4, ITGB6, KRT1, KRT10, KRT14, KRT2, KRT5, KRT9, LAMA3, LAMB2, LAMB3, LAMC1, LAMC2, MMP1, PKP1, PLCG2, PLEC, TGM5*	Italy	(27)

These early studies paved the way for subsequent studies, including the largest one to date, which utilized a targeted panel of 19 genes and demonstrated a high diagnostic yield with all its 138 patients having pathogenic mutations identified (20). The clinical utility of NGS targeted panel was compared with IFM alone, showing that IFM established EB subtypes in 76% (19 of 25) of cases, while 90% (36 of 40) were diagnosed by NGS (25). This is consistent with a recent retrospective analysis of 771 EB patients showing frequent equivocal findings with IFM compared to NGS (19). Overall, diagnostic yield ranged from 84 to 100% with three WES-based cohorts yielding genetic diagnoses in 95-100% of patients and six targeted sequencing cohorts yielding 84-100% (Table 1). Diagnostic yield for EB is among the highest for NGS among genetic conditions as seen in a study from the University of Minnesota assessing yield of gene panel testing of genetic disease across multiple specialties (28).

### Disorders of cornification – ichthyosis and PPK

Disorders of cornification are a category of genetic skin diseases characterized by xerosis, scaling, and/or hyperkeratosis due to abnormal keratinization. Inherited ichthyosis is the prototypical disease with 36 forms divided into syndromic and nonsyndromic forms (29). Nonsyndromic inherited ichthyoses are further subdivided into common ichthyoses, autosomal recessive congenital ichthyosis (ARCI), keratinopathic ichthyosis (KPI), and other. Up to 67 genes have been associated with ichthyosis and 28 genes with palmoplantar keratoderma (30–32).

Inherited ichthyosis and related disorders of cornification represent a diagnostic challenge due to heterogeneity and complex genotype–phenotype relationships. Mutations in different genes may produce similar phenotypes. This is exemplified by mutation screening in ARCI group patients for which 6 genes have been implicated, yet clinically are difficult to distinguish from one another due to overlap between subtypes. Meanwhile, mutations in one gene can also cause different subtypes of ichthyosis. Given the heterogeneity within ichthyoses, genetic testing has been particularly challenging due to the large number of associated genes. Various tests have been used to narrow candidate genes for genetic testing with Sanger sequencing. Traditionally, these include a combination of histopathology, transmission electron microscopy, and biochemical assays.

NGS has been applied in several large clinical cohorts (Table 2). The earliest targeted gene panel utilized microarray capture of probes across 24 genes in 14 patients (36). Of the 14 patients, 10 (71%) yielded pathogenic mutations, the majority of which were not previously reported. The largest study to date includes 1,000 genotyped ichthyosis patients from an international registry (30). In this large cohort, mutations were found in a total of 59 genes with description of 266 novel variants. When targeted sequencing failed to identify pathogenic variants, exome sequencing was performed, yielding a mutation in 87% of patients. The majority of patients were in the ARCI spectrum, among which severity of disease was associated with whether mutations were missense or nonsense.

**Table 2:** Ichthyosis cohorts with clinical NGS testing.

# of patients	Yield	Platform	Panel size (genes)	Region	Reference
1,000	87%	Targeted & WES	52 *AAGAB, ABCA12, ABHD5, ALDH3A2, ALOX12B, ALOXE3, AQP5, ATP2A2, ATP2C1, CARD14, CDSN, CERS3, CLDN1, CSTA, CYP4F22, DKC1, DSC2, DSG1, DSP, EBP, EDA, FLG, GJA1, GJB2, GJB3, GJB4, GJB6, KANK2, KRT1, KRT10, KRT16, KRT17, KRT2, KRT6C, KRT9, LOR, MBTPS2, NIPAL4, NSDHL, PNPLA1, POGLUT1, RHBDF2, RSPO1, SERPINB7, SLC27A4, SLURP1, SNAP29, SPINK5, SREBF1, STS, TGM1, TRPV3*	USA, Latin Am	(30)
64	83%	Targeted	37 *ABCA12, ABHD5, ALDH3A2, ALOX12B, ALOXE3, AP1S1, C7ORF11, CLDN1, CYP4F22, EBP, ERCC2, ERCC3, GBA, GJB2, GJB3, GJB4, GTF2H5, KRT1, KRT10, KRT2, LIPN, LOR, MBTPS2, NIPAL4, NSDHL, PEX7, PHYH, PNPLA1, POMP, SLC27A4, SNAP29, SPINK5, ST14, STS, SUMF1, TGM1, VPS33B*	Italy	(33)
45	79%	WES	40* ABCA12, ABHD5, ALOX12B, ALOXE3, AP1S1, CDSN, CERS3, CLDN1, CSTA, CYP4F22, DSG1, EBP, ELOVL4, ERCC2, ERCC3, FLG, GJB2, GJB3, GJB4, GJB6, GTF2H5, KRT1, KRT10, KRT2, KRT9, LIPN, LOR, MBTPS2, NIPAL4, PNPLA1, POMP, SLC27A4, SLURP1, SNAP29, SPINK5, ST14, STS, TGM1, TGM5, TRPV3	Norway	(34)
35	91%	Targeted	541 Includes following genes: *AAGAB, ABCA12, ABHD5, ALOX12B, ALOXE3, AP1S1, AQP5, ATP2A2, CDSN, CERS3, CLDN1, CSTA, CTSC, CYP4F22, DSG1, DSP, EBP, ELOVL4, ERCC2, ERCC3, FLG, GJB2, GJB3, GJB4, GJB6, GTF2H5, JUP, KRT1, KRT10, KRT14, KRT16, KRT17, KRT2, KRT6A, KRT6B, KRT9, LIPN, LOR, MBTPS2, NIPAL4, PKP1, PNPLA1, POMP, PTEN, RHBDF2, SDR9C7, SERPINB7, SLC27A4, SLURP1, SNAP29, SPINK5, ST14, STS, SULT2B1, TGM1, TGM5, TRPV, TRPV3, WNT10A*	China	(35)
14	71%	Targeted	24 *ABCA12, ABHD5, AP1S1, ALOXE3, ALOX12B, CDSN, CSTA, CYP4F22, DSG1, DSP, GJB2, GJB3, GJB4, KRT1, KRT2, LOR, NIPAL4, POMP, SLC27A4, SLURP1, SPINK5, STS, TGM1, TGM5*	United Kingdom	(36)
146 (ARCI)	83%	Targeted	38 *ABCA12, ABHD5, AGPS, ALDH3A2, ALOX12B, ALOXE3, AP1S1, ARSE, CERS3, CLDN1, CYP4F22, EBP, ELOVL4, GJB2, GJB3, GJB4, GJB6, KRT1, KRT10, KRT2, KRT9, LIPN, LOR, NIPAL4, PEX7, PHYH, PNPLA1, PNPLA2, POMP, SLC27A4, SNAP29, SPINK5, ST14, STS, TGM1, TGM5, VPS33B, ZMPSTE24*	United Kingdom	(37)
132 (ARCI)	85%	Targeted	79 Includes: *ABCA12, ABHD5, ALOX12B, ALOXE3, CERS3, CYP4F22, LIPN, NIPAL4, PNPLA1, SLC27A4, TGM1*	Denmark, Sweden	(38)
125 (ARCI)	79%	Targeted	38 *ABCA12, ABHD5, AGPS, ALDH3A2, ALOX12B, ALOXE3, AP1S1, ARSE, CERS3, CLDN1, CYP4F22, EBP, ELOVL4, GJB2, GJB3, GJB4, GJB6, KRT1, KRT10, KRT2, KRT9, LIPN, LOR, NIPAL4, PEX7, PHYH, PNPLA1, PNPLA2, POMP, SLC27A4, SNAP29,* *SPINK5, ST14, STS, TGM1, TGM5, VPS33B, ZMPSTE24*	Iran	(39)
34 (ARCI)	91%	Targeted	81 *ABCA12, ABHD5, ALDH3A2, ALOX12B, ALOXE3, AQP5, ATP2A2, BMP1, CALCR, CERS3, COL1A1, COL1A2, COL3A1, COL5A1, COL5A2, COL7A1, COL17A1, CRTAP, CTSC, CYP4F22, DSG1, DSP, DST, EDA, EDAR, EDARADD, EDN3, EDNRB, EXPH5, FBN1, FERMT1, FKBP10, GJB2, IKBKG, ITGA3, ITGA6, ITGB4, JUP, KRT1, KRT2, KRT5, KRT6A, KRT6B, KRT6C, KRT9, KRT10, KRT14, LAMA3, LAMB3, LAMC2, LEMD3, LEPRE1, LIPN, LOR, LRP5, MITF, NIPAL4, OCA2, PAX3, PDLIM4, PKP1, PLEC, PLOD1, PLOD2, PLS3, PNPLA1, PPIB, SERPINF1, SLC45A2, SNAI2, SOX10, SPINK5, STS, TGM1, TGM5, TNXB, TP63, TYR, TYRP1, VDR, WNT1*	Czech Republic	(40)
28 (ARCI)	79%	Targeted	13 *ABCA12, ALOX12B, ALOXE3, CASP14, CERS3, CYP4F22, LIPN, NIPAL4, PNPLA1, SDR9C7, SLC27A4, SULT2B1, TGM1*	India	(41)

*Only 40 ichthyosis-related genes were analyzed for variants of the WES data. ARCI, Autosomal recessive congenital ichthyosis.

Several other groups have utilized targeted sequencing panels specially for cohorts of ARCI group patients in United Kingdom, Denmark, Sweden, Iran, Czech Republic, and India (37–41). The diagnostic yield within these cohorts ranged from 79 to 91% with a mean yield of 84%. In the cases where mutations were not identified, one concern was the possibility of missed mutations being in genes outside of those included in the panels. Evidence of this was shown in a study of patients from Iran where the initial 38 gene panel yielded pathogenic mutations in 79% of patients, however when homozygosity mapping and Sanger sequencing of three additional genes more recently associated with ARCI were included, yield was further increased to 83% (39).

Panel sizes varied between studies ranging from 13 genes to 541 genes. Larger panels included genes associated with other genodermatoses not directly related to ichthyoses. Diagnostic yields generally were higher with larger panels, with cohorts showing the highest diagnostic yields being larger than 50 genes (30, 35, 38, 40). Compared to EB, yields were lower with larger panels indicating the greater heterogeneity in ichthyosis.

Palmoplantar keratodermas (PPKs) are a related group of conditions under disorders of cornification, characterized by hyperkeratosis of the palms and soles. One cohort of 64 patients with clinically diagnosed hereditary PPK were tested with either an in-house 35-gene NGS panel, a commercial NGS panel, or WES (42). Only 31 (48%) had a pathogenic mutation identified, with 21 (33%) having variants of unknown significance, and 12 (18%) with no suggestive variants identified.

## Squamous cell carcinoma

Cutaneous squamous cell carcinoma (cSCC) is the second most common cutaneous malignancy, comprising about 20% of all skin cancers (43). This results in roughly 1 million cases per year in the United States (44). Two to 5 % of cSCCs metastasize to lymph nodes or distant sites, and those that do have a worse prognosis (43, 45). In the US, cSCC is estimated to be responsible for 9,000 deaths per year (46). The mortality rate is estimated at 1-3 per 100,000 (47). The recent advances in deciphering the molecular biology of cSCC using NGS permits greater insight into pathogenesis and sets the stage for new diagnostic and therapeutic approaches (46, 48). For example, NGS has shown that cSCC is largely driven by mutations in tumor suppressor genes similar to other squamous cell carcinomas (49).

### Mutational landscape

The role of UV radiation has been shown to be central to the pathogenesis of cutaneous SCC, both in human and in animal models. Whole exome sequencing of cutaneous SCC and matched normal skin has shown that UV signature C-to-T transition base substitutions to be the most common mutational change in the tumors (50). In cases of squamous differentiation and more importantly in cases of undifferentiated histopathology, detection of the UV damage signature using NGS allows the identification of the source of Carcinoma of unknown primary (51). This has important clinical applications since cancer of unknown primaries account for 3-5% of all newly diagnosed advanced cancers (52).

*TP53* mutation is one of the first described and most established mutations in the pathogenesis of cSCC. Computational modeling using WES data showed that the loss of the second allele of *TP53* precedes other simple oncogenic mutations (50). Another study employing WES also found that acquisition of TP53 mutation promotes SCC *in-situ* (53). These findings further establish that early loss of *TP53* is an essential step of carcinogenesis in cSCC, similar to many other cancers such ovarian cancer, whether ensued from UV-induced DNA damage or other modes, confirming its driver role. *TP53* is the most frequently reported mutations in metastatic disease and is seen in ranges of 80-100% of patients (54–56).

Schwaederle et al. employed NGS to analyze over 200 genes in a large sample of SCC from different organ systems including the skin and found a common “squamousness gene signatures” consisting of *TP53*, *PIK3CA*, *CCND1*, *CDKN2A*, *SOX2*, *NOTCH1*, and *FBXW7* aberrations (57). They also made the interesting observation that *KRAS* alterations were absent in all types of SCC and that in cutaneous SCC specifically, p53 and Cyclin pathways and PIK3CA/SOX alterations were mutually exclusive. However, cSCC appears to partially differ in the presence of other driver mutations from that of other SCCs. Mutations in the oxidative stress gene *NFE2L2* and *PIK3CA* reported in lung and head and neck squamous cell carcinomas were rarely reported in cSCC (58). The anatomic location of cSCC is also associated with differences in genomic drivers. *KMT2C*, *KMTCD* and *PTCH1* are more common in periocular and eyelid cSCC (59). Human papillomavirus (HPV) infection has been linked to a large proportion of head and neck SCC and to the majority of genital and cervical SCC (60–62). cSCC has been shown to only rarely harbor HPV (63).

*CDKN2A* has been reported to be an early event in ocular surface and cutaneous SCC (63). It has also been reported in cSCC of the head and neck at a high frequency (64). *MYC* has been reported in precursor lesions along with *CCND1* and *EGFR* gains. NGS has shown that many DNA repair pathways are altered in cSCC. *PIK3C2B* mutations occur at a similar frequency in primary, recurrent and metastatic sSCC suggesting that these mutations are early events that may promote metastatic potential (65). *PIK3CA* have also been reported to be more common in locally advanced sSCC. *PIK3CG* mutation is common in metastatic cSCC (63).

The NOTCH family of receptors constitutes a conserved signaling pathway that has an essential role in epithelial cell fate determination such as proliferation and apoptosis (66). Mutations affecting the *NOTCH1* gene have been found to have differential roles in different human cancers. Activation of *NOTCH1*, whether through direct mutations or pathway activating mutations, are well established in the pathogenesis of lymphoblastic and lymphocytic leukemias (67, 68). On the other hand, *NOTCH1* and *NOTCH2* are genes that have been shown to have tumor suppression functions in human keratinocytes (69). *NOTCH1* and *NOTCH2* mutations have been reported to occur at high frequency in cSCC, shown in studies applying both WES and NGS panels among more than 200 patients combined (50, 54, 64, 70). WGS was used to discover a high frequency of *NOTCH1* and *NOTCH2* mutations in a cohort of 20 patients with advanced cSCC (71). Targeted deep sequencing was used to validate NOTCH mutations in 150 cases of cutaneous squamous neoplasia and confirmed NOTCH mutations in 82% of samples. South, et al. in this study also made the remarkable observation that *NOTCH1* mutations were present in precursor lesions as well. Through sequencing of adjacent and distant normal looking skin and correlation with western blotting and immunohistochemistry, they provided convincing evidence that *NOTCH1* is a main driver mutation occurring early in cSCC carcinogenesis, independent of *TP53* mutations. Zheng et al. confirmed these observations and showed that NOTCH mutations may precede *TP53* mutations in SCC *in-situ* by using WES. They also found that NOTCH loss-of-function mutations enriched in SCC *in-situ* differ from those in the adjacent epidermis. NOTCH mutations are also seen frequently in recurrent and metastatic cSCC and in immunocompromised hosts (49, 56, 65). Zilberg et al. also observed NOTCH1 mutations in non-metastatic cSCC but at a comparatively lower incidence than reported in metastatic cSCC in the literature (64), although their sample size was 10 patients.

Tumor mutational burden (TMB) is another consequence of UV-radiation unique to cutaneous SCC. The mutational burden in non-UV exposed squamous cell carcinoma of the penis is much lower, similar to head and neck and visceral squamous carcinomas (51). Despite the high mutational burden in cSCC, mutations leading to microsatellite instability such as *MLH1* are rare and exclusively seen in younger patients (72).

### Epigenetic alterations

Beyond dipyrimidine base substitutions mutations, UV radiation exerts epigenetic changes that directly promote carcinogenesis. Whole transcriptome and targeted RNA sequencing of clinical cSCC showed that *ID4*, a tumor suppressor gene, is downregulated by DNA methylation induced by UVB. The role of *ID4* methylation in promoting development of SCC was then elegantly confirmed using both animal models and *in-vitro* assays (73).

Precursor lesions to cSCC (actinic keratosis and SCC *in-situ*) have been shown to harbor recurrent somatic mutations and copy number changes almost identical to invasive cSCC. A key difference found using whole transcriptome sequencing was significant upregulation of genes promoting invasion including *MMP1*, *MMP3*, *MMP9*, *LAMC2*, *LGALS1*, and *TNFRSF12A* (63). These findings implicate alterations in epigenetic structure and machinery in promoting aggressive and metastatic behavior. Chromatin remodeling and histone modification seem to be shared among squamous cell carcinomas of different tissues of origin (74, 75). WES of primary cSCC and their corresponding metastasis allowed the discovery of *KMT2D* (MLL2) as a preferentially mutated gene in metastatic cSCC (56). *KMT2D* encodes a histone methyltransferase involved in chromatin remodeling and when mutated, it leads to genomic instability (76). In the same cohort, it was shown that other genes involved in epigenetic regulation including *KMT2C* (MLL3), *KMT2A*, *SETD2*, *EP300*, *KDM6A* and *CREBBP*, all previously reported in other visceral malignancies, were mutated in metastatic cSCC at higher rates. *KMT2D* (MLL2) and *KMT2C* (MLL3) have also been reported in primary cSCC (54).

### Predicting biologic behavior

Although the majority of cSCC are locally controlled by curative surgery, a subset presents as advanced disease or display aggressive biologic behavior with distant spread causing significant morbidity and mortality. Although clinical and pathological staging is used to stratify patients’ risk and guide therapy, it may not fully capture the risk of aggressive behavior in some early-stage tumors while overestimating the risk of other advanced tumors, as is the case with other cancer (77). For example, tumor thickness has been shown to be the single most significant predictor of metastasis (78). On the other hand, differentiation has often failed to correlate with disease outcome (65).

PI3K/AKT signaling pathway correlates with E-cadherin to N-cadherin expression, a step in epithelial-to-mesenchymal transition that may facilitate metastasis (79). Oncogenic alterations activating the RAS/RTK/PI3K pathway have been reported to be prevalent in approximately half of cSCCs from the head and neck region with lymph nodes metastasis and correlate with a worse progression-free survival (64). Genes implicated in epigenetic regulation such as the KMT2 family have been observed in metastatic disease more frequently. *MSH6* mutation in periocular and eyelid cSCC carries a higher risk of nodal and distant metastasis in periocular and eyelid cSCC (59). DNA repair genes may also serve as a marker for aggressive disease. A systematic review by Lobl et al. found that *P53*, *TERT*, *SPEN*, *MLL3*, and *NOTCH2* mutations were significantly more likely to be found in metastatic versus localized SCCs (46). Lobl et al. noted less mutation concordance between primary and metastatic tumors in immunosuppressed patients supporting that the loss of the anti-tumor immune response promoted metastasis by the loss of immune editing (48). CD274 (PD-L1) amplification was rare in metastatic penile SCC and cSCC (51). Copy number alterations in the 3q chromosome may predict response to immune checkpoint inhibition (65).

Despite the significant insight into the biology of cSCC and potential future applications for prognosis and therapies, current studies have displayed several limitations. Most studies only have 30-40 patients, few studies conducted WES or WGS analyzes, and the source of tissue was also almost always formalin-fixed paraffin-embedded tissue. Almost all studies only sampled the cancer once to obtain genetic materials, which increases the risk of bias given presence of intertumoral heterogeneity. Future studies should focus on amending these limitations and include larger numbers of participants to improve the generalizability and clinical relevance of findings.

## Melanoma

Melanoma is a malignant tumor of pigment-forming melanocytes. It is an aggressive cutaneous malignancy, making up 1% of all skin cancers yet accounting for the majority of skin cancer deaths (80). The genetic underpinning of melanoma was established by identification of somatic *BRAF* and germline *CDKN2A* mutations in cutaneous melanoma and familial melanoma, respectively (81–83). *BRAF* is one of the most frequently mutated genes in melanoma, with rates ranging from 20 to 80%, and the hotspot V600E mutation accounting for 60-80% of BRAF mutations (81, 84). Other somatic mutations have also since been identified in melanomas including *TERT*, *NRAS*, *NF1*, and *KIT* in approximately 70-85%, 20-30%, 10-15, and 10% of melanomas, respectively (84–87). Melanomas have traditionally been classified based on histologic type and anatomic location, including superficial spreading melanoma, nodular melanoma, lentigo maligna melanoma, acral lentiginous melanoma, and uveal melanoma. Genomic analysis has demonstrated variation in frequency of somatic mutations differing based on subtype. More recently, classification into nine distinct melanoma evolutionary pathways have been developed based on histologic, clinical, and epidemiological features (88, 89). Somatic *BRAF* mutations are found most frequently in skin with low cumulative solar damage (CSD), which predominately present as superficial spreading melanomas. Meanwhile, melanomas arising in high CSD skin, which present as lentigo maligna melanomas, contain more *NRAS* and *KIT* mutations. Acral melanomas harbor *KIT*, *NRAS*, and *BRAF* mutations, mucosal melanomas *KIT* and *NRAS* mutations, and uveal and melanomas arising in blue nevi uniquely have *GNA11* and *GNAQ* mutations.

Identification of these mutations have resulted in the use of targeted therapies, such as the BRAF inhibitor vemurafenib in melanomas with V600E mutation, which was approved in 2011 (90). Sequencing has been used to detect these mutations, traditionally by real-time PCR-based techniques such as the FDA approved cobas 4,800 BRAF V600 mutation test, which is approved as a companion diagnostic for vemurafenib and cobimetinib. Next-generation sequencing represents a powerful tool that can take advantage of the broadening mutational landscape and is increasingly used in the clinic in the management of melanomas.

While large-scale whole-genome and whole-exome sequencing were used in identifying and cataloging mutations in melanoma, these techniques are impractical for routine use given cost and excessive data requiring customized bioinformatic analysis. Targeted sequencing panels utilize DNA capture technology to select particular genes and genomic loci to resequence. Compared to traditional PCR-based tests, which are designed to test small portions of single genes, sequencing panels can cover the entire span of a gene as well as many genomic loci in parallel. Targeted sequencing represents a middle ground between PCR-based testing of individual loci and whole genome and exome-based comprehensive testing. This allows for efficient testing focused on genes known to be important in disease and actionable targets for therapeutics and has become the preferred molecular test for melanoma.

### Next-generation sequencing panels for somatic mutations in melanoma

Many NGS gene panels with various designs have been developed and tested in the past decade. The utility of these panels is exhibited by the high yields, with 70 to 92% of tested melanomas identifying one or more pathogenic mutations (Table 3) (94, 96). Among identified mutations, a large number are actionable with management implications. One study utilizing a panel of 248 genes found that 16 of among 18 patient-derived tumors samples (89%) had actionable mutations including those in *BRAF*, *ALK*, *ERBB4*, *KIT*, and *PIK3CA* (98). Similarly, in a cohort of 36 melanomas from Korea, 92% had an alteration detected and 70% of patients had actionable alterations, which were amenable to treatment with standard or investigational drugs (96). Real-world assessment of actionability showed that melanomas had the highest frequency of actionable alterations among 49 cancer types with 28 of 37 (76%) melanomas harboring actionable alterations based on the OncoKB database (103). Many of these mutations allowed enrollment of patients into early phase trials targeted toward mutations identified via sequencing panels.

**Table 3:** Melanoma cohorts with clinical NGS testing.

# of samples	Population	Yield	Panel size (genes)	Reference
699	Cutaneous melanoma (including acral), mucosal melanoma, uveal melanoma; United States (somatic)	556/699 (80%)	46 *ABL1, AKT1, ALK, APC, ATM, **BRAF**, CDH1, CDKN2A, CSF1R, CTNNB1, EGFR, ERBB2, ERBB4, FBXW7, FGFR1, FGFR2, FGFR3, FLT3, GNAS, HNF1A, HRAS, IDH1, JAK2, JAK3, KDR, KIT, KRAS, MET, MLH1, MPL, NOTCH1, NPM1, **NRAS**, PDGFRA, PIK3CA, PTEN, PTPN11, RB1, RET, SMAD4, SMARCB1, SMO, SRC, STK11, **TP53**, VHL*	(91)
132 patients	Cutaneous (including acral lentiginous melanoma, melanoma in blue nevus), uveal melanoma, mucosal melanoma; United Kingdom (somatic)	93/132 (70%)	7 ***BRAF**, GNA11, **GNAQ**, KIT, KRAS, MAP2K1, **NRAS***	(92)
121	Cutaneous melanoma; United States (somatic)	104/121 (86%)	50 *ABL1, AKT1, ALK, APC, ATM, **BRAF**, CDH1, **CDKN2A**, CSF1R, CTNNB1, EGFR, ERBB2, ERBB4, EZH2, FBXW7, FGFR1, FGFR2, FGFR3, FLT3, GNA11, GNAQ, GNAS, HNF1A, HRAS, IDH1, IDH2, JAK2, JAK3, KDR, KIT, KRAS, MET, MLH1, MPL, NOTCH1, NPM1, **NRAS**, PDGFRA, PIK3CA, PTEN, PTPN11, RB1, RET, SMAD4, SMARCB1, SMO, SRC, STK11, TP53, VHL*	(93)
100	Cutaneous melanomas; Spain (somatic)	85/100 (85%)	35 *ADAMST18, ALK, BPA1, **BRAF**, CDK4, CDKN2A, EPHA7, ERBB4, GNA11, GNAQ, GRIN2A, GRM3, HOXD8, HRAS, IRS4, KIT, KRAS, MAP2K1, MAP2K2, MC1R, MET, MITF, NF1, **NRAS**, PIK3CA, PPP6C, **PREX2**, PTEN, RAC1, STK11, STK19, STK31, TAF1L, TERT, TRRRAP*	(94)
71	Mucosal melanomas; Germany (somatic)	50/71 (70%)	29 *ARID1A, ARID2, BAP1, **BRAF**, CDK4, CDKN2A, CTNNB1, EZH2, FBXW7, GNA11, GNAQ, HRAS, IDH1, KIT, KRAS, MAP2K1, MAP2K2, MITF, **NF1**, **NRAS**, PIK3CA, PIK3R1, PTEN, RAC1, SF3B1, SMARCA4, TERT, TP53, WT1*	(95)
36	*BRAF* wild-type recurred or metastatic melanoma (acral); Korea (somatic)	33/36 (92%)	225 *ABL1, ABL2, AKT1, AKT2, AKT3, ALK, APC, AR, ARAF, ARID1A, ATM, ATR, AURKA, AURKB, AURKC, AXL, BAP1, BARD1, BCL2, BRAF, BRCA1, BRCA2, BRIP1, BTK, CBFB, CBL, CCND1, CCND2, CCND3, CCNE1, CDH1, CDK1, CDK4, CDK6, CDK11B, CDK12, CDKN1A, CDKN1B, **CDKN2A**, CDKN2B, CDKN2C, CEBPA, CHEK1, CHEK2, CREBBP, CSF1R, CTNNB1, DDR1, DDR2, DICER1, DNMT3A, DOT1L, DPYD, EGFR, EIF1AX, EMSY, EP300, EPCAM, EPHA3, ERBB2, ERBB3, ERBB4, ERCC2, ERG, ESR1, ETV1, EWSR1, EZH2, FAM175A, FANCA, FANCC, FANCD2, FANCG, FANCI, FANCL, FANCM, FBXW7, FGF3, FGF4, FGF6, FGF10, FGF14, FGF19, FGF23, FGFR1, FGFR2, FGFR3, FGFR4, FLT1, FLT3, FLT4, FOXA1, FOXL2, GNA11, GNAQ, GNAS, GNB2L1, HDAC1, HDAC9, HGF, HRAS, IDH1, IDH2, IGF1R, IGF2, IGFBP3, INPP4B, IRF1, JAK1, JAK2, JAK3, JUN, KDM5C, KDM6A, KDR, KEAP1, **KIT**, KMT2D, KRAS, LATS1, LATS2, MAP2K1, MAP2K2, MAP2K4, MAP3K1, MAP3K4, MAPK1, MAPK8, MCL1, MDM2, MDM4, MET, MLH1, MPL, MRE11A, MSH2, MSH6, MTOR, MUTYH, MYC, MYCN, NEK2, NF1, NF2, NFE2L2, NOTCH1, NOTCH2, NOTCH3, NOTCH4, NPM1, **NRAS**, NRG1, NTRK1, NTRK2, NTRK3, NUTM1, PAK2, PALB2, PARP1, PARP2, PBRM1, PDGFB, PDGFRA, PDGFRB, PIK3CA, PIK3CB, PIK3CD, PIK3R1, PIK3R2, PMS2, POLD1, POLE, POLQ, PPARG, PPP2R2A, PRKCB, PTEN, RAD21, RAD50, RAD51, RAD51B, RAD51C, RAD51D, RAD54L, RB1, RBM10, RELA, RET, RHEB, RICTOR, RIT1, RNF43, ROS1, RPTOR, RSPO1, SDHB, SDK1, SETD2, SMAD4, SMARCA4, SMARCB1, SMG1, SOX2, SPOP, SQSTM1, SRC, SS18, STAT1, STAT6, STK11, SUMO1, SYK, TERT, TFE3, TOP2A, TP53, TP63, TPMT, TSC1, TSC2, TSHR, UGT1A1, VHL, XRCC2, ZBTB16*	(96)
30	Melanoma metastases; United States (somatic)	30/30 (100%)	182 *ABL1, ABL2, AKT1, AKT2, AKT3, ALK, APC, AR, ARAF, ARFRP1, ARID1A, ATM, ATR, AURKA, AURKB, BAP1, BCL2, BCL2A1, BCL2L1, BCL2L2, BCL6, **BRAF**, BRCA1, BRCA2, CARD11, CBL, CCND1, CCND2, CCND3, CCNE1, CD79A, CD79B, CDH1, CDH2, CDH5, CDH20, CDK4, CDK6, CDK8, CDKN2A, CDKN2B, CDKN2C, CEBPA, CHEK1, CHEK2, CRKL, CRLF2, CTNNB1, DDR2, DNMT3A, DOT1L, EGFR, EPHA3, EPHA5, EPHA6, EPHA7, EPHB1, EPHB4, EPHB6, ERBB2, ERBB3, ERBB4, ERCC2, ERG, ESR1, EZH2, FANCA, FBXW7, FGFR1, FGFR2, FGFR3, FGFR4, FLT1, FLT3, FLT4, FOXP4, GATA1, GNA11, GNAQ, GNAS, GPR124, GUCY1A2, HOXA3, HRAS, HSP90AA1, IDH1, IDH2, IGF1R, IGF2R, IKBKE, IKZF1, INHBA, INSR, IRS2, JAK1, JAK2, JAK3, JUN, KDM6A, KDR, KIT, KRAS, LRP1B, LRP6, LTK, MAP2K1, MAP2K2, MAP2K4, MCL1, MDM2, MDM4, MEN1, MET, MITF, MLH1, MLL, MPL, MRE11A, MSH2, MSH6, MTOR, MUTYH, MYC, MYCL1, MYCN, **NF1**, NF2, NKX2-1, NOTCH1, NPM1, NRAS, NTRK1, NTRK2, NTRK3, PAK3, PAX5, PDGFRA, PDGFRB, PHLPP2, PIK3CA, PIK3CG, PIK3R1, PKHD1, PLCG1, PRKDC, PTCH1, PTCH2, PTEN, PTPN11, PTPRD, RAF1, RARA, RB1, RET, RICTOR, RPTOR, RUNX1, SMAD2, SMAD3, SMAD4, SMARCA4, SMARCB1, SMO, SOX2, SOX10, SRC, STAT3, STK11, SUFU, TBX22, TET2, TGFBR2, TNFAIP3, TNKS, TNKS2, TOP1, **TP53**, TSC1, TSC2, USP9X, VHL, WT1*	(97)
18	Cutaneous melanoma (metastatic); United States (somatic)	16/18 (89%)	248 *ABCB1, ABL1, ABL2, ADRB1, ADRB2, AKT1, AKT2, AKT3, ALK, ALOX5, APC, APC2, AR, ARID1A, ARID1B, ARID2, ARID3A, ARID3B, ARID4A, ARID4B, ARID5A, ARID5B, ASXL1, ATM, ATR, AURKA, BCL2, BCR, **BRAF**, BRCA1, BRCA2, BRD4, CBL, CBLB, CCND1, CCNE1, CDC73, CDH1, CDH6, CDK4, CDK6, CDK8, CDKN1A, CDKN1B, CDKN2A, CDKN2B, CEBPA, CHD5, CHEK1, CHEK2, COBRA1, COMT, CREBBP, CRKL, CSF1R, CTNNB1, CYP2A6, CYP2B6, CYP2C8, CYP2C9, CYP2C19, CYP2D6, CYP3A4, CYP3A5, CYP4F2, DNMT3A, DPYD, DRD2, EGFR, EPHA3, EPHA5, EPHA6, EPHA10, EPHB6, ERBB2, ERBB3, ERBB4, ERCC1, ERG, FAM123B, FBXW7, FGFR1, FGFR2, FGFR3, FGFR4, FHIT, FKBP9, FLT1, FLT3, FLT4, FOLR1, G6PD, GATA3, GNA11, GNAQ, GNAS, GSTM1, GSTP1, GSTT1, GUCY1A2, H3F3A, H3F3B, HECW1, HLA-A, HLA-B, HNF1A, HRAS, HSP90AA1, IDH1, IDH2, IGF1R, IKBKE, IKZF1, IL28B, ITPA, JAK1, JAK2, JAK3, JARID2, KCNH2, KCNJ11, KDM5A, KDM5B, KDM5C, KDM6A, KDR, KEAP1, **KIT**, KRAS, MAP2K1, MAP2K2, MAP2K4, MAP3K1, MAP3K8, MCL1, MDM2, MDM4, MEN1, MERTK, MET, MITF, MLH1, MLL, MLL2, MLL3, MPL, MRE11A, MSH2, MSH6, MTF2, MTHFR, MTOR, MTUS2, MYC, MYCL1, MYCN, MYD88, NAT2, NF1, NF2, NFE2L2, NKX2-1, NOTCH1, NOTCH2, NOTCH3, NOTCH4, NPM1, NQO1, **NRAS**, NTRK1, NTRK2, NTRK3, PAK7, PAX5, PBRM1, PDGFRA, PDGFRB, PDPK1, PHF6, PHF19, PIK3CA, PIK3CD, PIK3R1, PIM1, PLK1, PRKDC, PTCH1, PTEN, PTK2, PTK2B, PTPN11, PTPRD, RAF1, RB1, REL, RET, RICTOR, ROR2, ROS1, RPTOR, RRM1, RUNX1, RUNX1T1, SCN5A, SETD2, SETDB1, SLCO1B1, SMAD2, SMAD3, SMAD4, SMARCA4, SMARCB1, SMO, SOCS1, SPEN, SRC, STAT3, STK11, SUFU, SULT1A1, SUPT4H1, SUPT5H, TCF3, TCF4, TERT, TET1, TET2, TGFBR2, TMPRSS2, TNFAIP3, TNK2, TOP1, TOP2A, TP53, TPMT, TSC1, TSC2, TSHR, TYK2, TYMS, UGT1A1, UTY, VHL, VKORC1, WHSC2, WT1, ZNF668*	(98)
15	Anorectal melanoma; United States (somatic)	14/15 (93%)	467 *ABL1, AKT1, AKT2, AKT3, ALK, ALOX12B, AMER1, APC, AR, ARAF, ARID1A, ARID1B, ARID2, ARID5B, ASXL1, ASXL2, ATM, ATR, ATRX, AURKA, AURKB, AXIN1, AXIN2, AXL, B2M, BAP1, BARD1, BBC3, BCL2L1, BCL2L11, BCL6, BCL11B, BCOR, BCORL1, BCR, BLM, BMPR1A, BRAF, BRCA1, BRCA2, BRCC3, BRD3, BRD4, BRIP1, BTK, BUB1B, CALR, CARD11, CASP8, CBL, CBLB, CBLC, CCND3, CCNE1, CD58, CD74, CD79A, CD79B, CD274, CD276, CDC6, CDC7, CDC45, CDC73, CDCA5, CDH1, CDK4, CDK6, CDK8, CDK12, CDKN1A, CDKN1B, CDKN2A, CDKN2B, CDKN2C, CDT1, CEBPA, CHEK1, CHEK2, CIC, CIITA, CLTC, CLTCL1, CNOT3, CREBBP, CREBBP, CRKL, CRLF2, CRLF2, CSF1R, CSF3R, CTCF, CTLA4, CTNNB1, CUL3, CYLD, DAXX, DCUN1D1, DDB2, DDR2, DICER1, DIS3, DNM2, DNMT1, DNMT3A, DNMT3B, DOT1L, E2F3, ECT2L, EED, EGFL7, EGFR, EIF1AX, EP300, EPCAM, EPHA3, EPHA5, EPHB1, ERBB2, ERBB3, ERBB4, ERCC2, ERCC3, ERCC4, ERCC5, ERG, ESR1, ETV1, ETV4, ETV5, ETV6, EWSR1, EXT1, EXT2, EZH2, EZR, FAM46C, FAM175A, FANCA, FANCC, FANCD2, FANCE, FANCF, FANCG, FAS, FAT1, FBXO11, FBXW7, FGF3, FGF4, FGF19, FGFR1, FGFR2, FGFR3, FGFR4, FH, FIP1L1, FLCN, FLT1, FLT3, FLT4, FOXA1, FOXL2, FOXO1, FUBP1, FUS, FYN, GATA1, GATA2, GATA3, GMNN, GNA11, GNA13, GNAQ, GNAS, GNB1, GOPC, GPC3, GREM1, GRID1, GRIN2A, GSK3B, H3F3A, H3F3C, HGF, HIST1H1C, HIST1H2BD, HIST1H3B, HMGA2, HNF1A, HRAS, ICOSLG, ID3, IDH1, IDH2, IFNGR1, IGF1, IGF1R, IGF2, IKBKE, IL2, IL6ST, IL7R, IL10, INPP4A, INPP4B, INSR, IRF1, IRF4, IRF8, IRS1, IRS2, ITK, JAK1, JAK2, JAK3, JUN, KAT6A, KCNJ5, KDM5C, KDM6A, KDM6B, KDR, KEAP1, KIAA1549, KIF5B, **KIT**, KLF4, KLF6, KLHL6, KMT2A, KMT2C, KMT2D, KRAS, LAMB4, LATS1, LATS2, LMO1, LRIG3, LUC7L2, MAP2K1, MAP2K2, MAP2K4, MAP3K1, MAP3K13, MAPK1, MAX, MCL1, MCM2, MCM3, MCM4, MCM5, MCM6, MCM7, MDC1, MDM2, MDM4, MECOM, MED12, MEF2B, MEN1, MET, MITF, MLH1, MLLT10, MPL, MRE11A, MSH2, MSH6, MTOR, MUTYH, MYC, MYCL, MYCN, MYD88, MYOD1, NBN, NCOR1, **NF1**, NF2, NFE2L2, NIPBL, NKX2-1, NKX3-1, NOTCH1, NOTCH2, NOTCH3, NOTCH4, NPM1, NRAS, NT5C2, NTRK1, NTRK2, NTRK3, NUP98, NUP214, NUTM1, PAK1, PAK7, PALB2, PARK2, PARP1, PAX5, PAX8, PBRM1, PDCD1, PDGFRA, PDGFRB, PDPK1, PHF6, PHF8, PHOX2B, PICALM, PIGA, PIK3C2G, PIK3C3, PIK3CA, PIK3CB, PIK3CD, PIK3CG, PIK3R1, PIK3R2, PIK3R3, PLAG1, PLK2, PMAIP1, PML, PMS1, PMS2, PNRC1, POLE, POT1, PPARG, PPP2R1A, PRDM1, PRF1, PRKAR1A, PRPF8, PRPF40B, PTCH1, PTEN, PTPN1, PTPN11, PTPRC, PTPRD, PTPRS, PTPRT, PTTG1, RAC1, RAD21, RAD50, RAD51, RAD51B, RAD51C, RAD51D, RAD52, RAD54L, RAF1, RARA, RASA1, RB1, RBM10, RECQL4, REL, RET, RFWD2, RHOA, RICTOR, RIT1, RNF43, ROS1, RPL5, RPL10, RPS6KA4, RPS6KB2, RPTOR, RUNX1, RYBP, SBDS, SDHA, SDHAF2, SDHB, SDHC, SDHD, SETBP1, SETD2, SF1, SF3A1, **SF3B1**, SH2B3, SH2D1A, SHQ1, SLC45A3, SMAD2, SMAD3, SMAD4, SMARCA4, SMARCB1, SMARCD1, SMARCE1, SMC1A, SMC3, SMO, SOCS1, SOX2, SOX9, SOX17, SPEN, SPOP, SRC, SRSF2, SS18, SSX1, SSX2, SSX4, STAG1, STAG2, STAG3, STAT3, STAT5B, STAT6, STK11, STK40, SUFU, SUZ12, SYK, TAF15, TBL1XR1, TBX3, TCF3, TCF12, TERT, TET1, TET2, TET3, TFE3, TGFBR1, TGFBR2, TMEM127, TMPRSS2, TNFAIP3, TNFRSF14, TOPBP1, **TP53**, TP53BP1, TP63, TPM3, TRAF7, TSC1, TSC2, TSHR, U2AF1, U2AF2, UBR5, USP6, VHL, VTCN1, WAS, WRN, WT1, XIAP, XPA, XPC, XPO1, YAP1, YES1, ZRSR2*	(99)
451 families	Patients with cutaneous and uveal melanomas who had family history of melanoma, but no *CDKN2A* or *CDK4* mutations; Netherlands (germline)	18/451 (4%)	30 *ACD, **BAP1**, BRIP1, CBLB, CDK4, CDKN2A, CENPS, CREB3L1, DOT1L, ERCC3, MC1R, **MITF**, MLLT6, NEK10, NEK11, NEK2, NEK4, OCA2, PARP1, POLE, POLH, POT1, PTEN, RAD51B, RASEF, TERF1, TERF2IP, TERF2IP, TERT, TINF2*	(100)
264	High risk melanoma patients with family history, other cancers, multiple primary melanomas, or early onset; Czech Republic (germline analysis of peripheral blood)	43/264 (16%)	217 *ABLIM1, ACD, AGR3, APC, APEX1, ARNT, ASIP, ATM, ATRN, AURKA, BAP1, BARD1, BBC3, BLM, BMPR1A, BRAF, BRCA1, BRCA2, BRIP1, BRMS1, CASP8, CASP10, CBL, CCAR2, CCND1, CCNH, CDH1, CDK4, CDK7, CDK10, CDKN1A, CDKN1B, CDKN1C, **CDKN2A**, CDKN2B, CEBPA, CHEK2, CLPTM1L, COX8A, CTLA4, CTNNB1, CYP1A1, CYP1A2, CYP3A5, CYP11A1, CYP17A1, CYP19A1, DAB2IP, DCAF4, DDB1, DDB2, EDNRB, EGF, EGFR, EIF1AX, EPCAM, ERBB2, ERBB4, ERCC1, ERCC2, ERCC3, ERCC4, ERCC5, ERCC6, ERCC8, EXOC2, EZH2, FANCC, FANCL, FANCM, FAS, FASLG, FGFR2, FGFR4, FH, FLCN, FLT1, FOXP3, FTO, GATA2, GATA4, GC, GNA11, GNAQ, GPC3, GSTM1, GSTM3, GSTP1, GSTT1, H2AFY, HERC2, HRAS, IDH1, IDH2, IFIH1, IFNA1, IFNG, IL2RA, IL4, IL6, IL8, IL10, ING4, IRF4, KAT6A, KIAA1967, KIT, KMT2A, KRAS, LRIG1, MAP2K1, MC1R, MDM2, MET, MGMT, MITF, MLH1, MLH3, MMP1, MMP3, MSH2, MSH3, MSH6, MTAP, MUTYH, MX2, MYH7B, **NBN**, NCOA6, NF1, NF2, NFKB1, NFKBIE, NOD2, NOTCH3, NRAS, OBFC1, **OCA2**, PALB2, PARP1, PAX5, PDGFRA, PIGU, PIK3CA, PIK3R1, PIK3R4, PLA2G6, PMAIP1, PMS1, PMS2, POLD1, POLE, POLH, POMC, POT1, PPM1D, PPP6C, PRF1, PTCH1, PTEN, PTGS2, PTPN11, PTPN22, RAC1, RAD23A, RAD23B, RAD51C, RAD51D, RASEF, RB1, RECQL, RECQL4, RET, RHOBTB2, RUNX1, SBDS, SDHA, SDHB, SDHC, SDHD, SETDB1, SF3B1, SH2B3, SLC24A4, SLC45A2, SLX4, SMAD4, SMARCB1, SNX31, STAG2, STK11, STK19, SUZ12, TACC1, TERC, TERF1, TERF2, TERF2IP, TERT, TINF2, TLR3, TP53, TRPM1, TSC1, TSC2, TYR, TYRP1, VDR, VHL, WRN, WT1, XAB2, XPA, XPC, XRCC1, XRCC3, ZNF365*	(101)
102	Melanoma patients with multiple primary melanomas; Italy (germline analysis of peripheral blood)	76/102 (75%)	29 *ACD, AGR3, ARNT, ASIP, **ATM**, **BAP1**, CASP8, CCND1, CDK4, CDKAL1, CDKN2A, FTO, GC, IRF4, **MC1R**, MITF, MX2, OBFC1, OCA2, PALB2, PARP1, POT1, RMND2, SLC45A2, TERF21P, TERT, TMEM38B, TYR, TYRP1*	(102)

Bolded: Top three mutated genes.

There is heterogeneity with regards to the number of genes and which melanoma genes are included among gene sequencing panels. One retrospective analysis comparing five separate NGS panels found sizes ranging from 50 to 400 genes, with only 23 overlapping genes between the five panels (104). Among our review of the literature, number of genes varied from as few as 7 to as many as 467 genes between studies (Table 3) (92, 99). Often, larger panels exist as general cancer sequencing panels that are used across multiple cancer types and contain general oncogenes not prevalent in melanoma.

The exact composition of the NGS panels vary between groups and play a role in determining sensitivity of the test, particularly for melanoma at special sites. For example, melanomas from acral, mucosal, and uveal sites have been shown to harbor unique mutations. Mutations in *KIT* are enriched at acral and mucosal sites, while mutations in *GNAQ* and *GNA11* are increased among uveal melanomas (86, 105). This was demonstrated in one study with a large cohort of 699 patients, including acral, mucosal, and uveal melanomas sequenced with a 46 gene pan-cancer NGS panel (91). The authors noted a high rate of acral, mucosal, and uveal melanomas with no detected mutations, 33, 44, and 92% respectively, compared to 15% of cutaneous melanomas. This is thought to be due to omission of subtype-specific genes such as *GNAQ*, *GNA11*, and *BAP1* in the panel, decreasing sensitivity of the test. This particular panel also excluded genes such as *TERT*, *NF1*, and *RAC1*, which were contemporaneously identified, further decreasing sensitivity (85, 87).

The current state of NGS sequencing panels has matured with increasing number of commercial panels, some of which have obtained FDA approval. These panels have been developed to capture many gene targets across different cancer types, so called “pan-cancer” panels. Three panels are FDA approved and have been tested on melanoma specimen: MSK-IMPACT, FoundationOne CDx, and PGDx elio tissue complete. The MSK-IMPACT targeted sequencing panel consisting of 468-gene was approved by the FDA in 2017 for tumor profiling and not as a companion diagnostic to any medication (106). Studies utilizing the MSK-IMPACT panel show melanoma to be among the most actionable among various cancers, with rates of actionable mutations ranging from 58 to 76% of clinical samples (103, 107). Similarly, the FoundationOne panel was initially developed with 287 genes and has undergone changes in the panel leading to 324 gene panel approved by the FDA in 2017 as a companion diagnostic for 15 different targeted therapies including BRAF or BRAF/MEK inhibitor combinations (108). The FoundationOne panel in a study of 30 metastatic melanoma cases showed clinically relevant genomic alterations in all patients (Table 3) (97). Lastly, Personal Genome Diagnostics’ PDGx elio tissue complete was approved in 2020 containing 505 genes and an automated bioinformatic analysis platform. The platform was validated using a pan-solid tumor sample including 455 melanomas showing high accuracy and concordance for sequence alterations, structural variants, and tumor mutation burden (109).

### Next-generation sequencing panels for detection of germline melanoma susceptibility genes

The application of NGS sequencing panels in melanoma also extends to patients at high-risk of developing multiple melanomas due to the presence of germline mutations in melanoma susceptibility genes. *CDKN2A*, which encodes p16(INK4A) and p14(ARF) cell cycle-related tumor suppressors, was the first familial susceptibility gene and among the most highly penetrant with 30-90% risk of melanoma by age 80 years. Other high-to-moderate risk genes include *CDK4*, *BAP1*, *TERT*, *POT1*, *MITF*, *TERF2IP*, and *ACD* (101).

Several studies have utilized NGS panels to investigate the presence of melanoma susceptibility genes among melanoma patients with risk factors (Table 3). Diagnostic yield among ranged widely from 4 to 75%. In one study of 264 Czech melanoma patient indicated for genetic testing due to presence early melanomas (<25 years old), presence of multiple primary melanomas or other cancers in their personal or family history, 71/254 (27%) of patients had a pathogenic or likely pathogenic germline variant identified, 43/264 (16%) carried a mutation in a gene associated with melanoma or other cancer, 9/264 (3%) carried clinically important high-to-moderate melanoma risk genes (*CDKN2A*, *POT1*, *ACD*), and 22/264 (8%) in other cancer syndrome genes (*NBN*, *BRCA1/2*, *CHEK2*, *ATM*, *WRN*, *RB1*) (101). In a separate study of 451 families with no germline *CDKN2A* or *CDK4* mutations, the diagnostic yield was low with only 18/451 (4%) families having pathogenic variants (100). Lastly, an Italian study reported higher diagnostic yield with 76/102 (75%) of patients having at least one pathogenic mutation in *MC1R*, *ATM*, *BAP1*, *CDKN2A*, *PALB2*, or *TYR* (102). This difference is attributed to inclusion of *MC1R*, a low-risk susceptibility gene responsible for pigmentary regulation, as well as the cohort consisting of patients with multiple primary melanomas rather than with family history. Such targeted panels are available as commercial clinical NGS tests for melanoma that are intended for germline testing of susceptibility genes (Table 4). These panels are anticipated to continue to grow as the compendium of known deleterious variants expands and is better characterized.

**Table 4:** Commercial NGS testing companies and clinical tests available in the United States.

Company	Genodermatoses		Melanoma susceptibility
	EB	Ichthyosis	
GeneDx	28 *CD151, CDSN, CHST8, COL17A1, COL7A1, CSTA, DSG1, DSP, DST, EXPH5, FERMT1, FLG2, ITGA3, ITGA6, ITGB4, JUP, KLHL24, KRT1, KRT10, KRT14, KRT5, LAMA3, LAMB3, LAMC2, PKP1, PLEC, SERPINB8, TGM5*	49 *ABCA12, ABHD5, ALDH3A2, ALOX12B, ALOXE3, AP1S1, ARSL (ARSE), CASP14, CDSN, CERS3, CHST8, CLDN1, CSTA, CYP4F22, EBP, ELOVL4, FLG, FLG2, GJB2, GJB3, GJB4, GJB6, KDSR, KRT1, KRT10, KRT2, KRT9, LIPN, LOR, MBTPS2, NIPAL4, NSDHL, PEX7, PHGDH, PHYH, PNPLA1, POMP, PSAT1, SDR9C7, SERPINB8, SLC27A4, SNAP29, SPINK5, ST14, STS, TGM1, TGM5, VPS33B, ZMPSTE24*	9 *BAP1, BRCA2, CDK4, CDKN2A, MITF, POT1, PTEN, RB1, TP53*
Fulgent	13 *CD151, COL17A1, COL7A1, DSP, ITGA3, ITGB4, KRT14, KRT5, LAMA3, LAMB3, LAMC2, MMP1, PLEC*	44 *ABCA12, ABHD5, ALDH3A2, ALOX12B, ALOXE3, AP1S1, ARSE, CASP14, CERS3, CLDN1, CRYL1, CYP4F22, EBP, ELOVL4, ERCC2, ERCC3, FLG, GJA1, GJB2, GJB3, GJB4, GJB6, GTF2H5, KRT1, KRT10, KRT2, KRT9, LIPN, LOR, MPLKIP, NIPAL4, PEX7, PHYH, PNPLA1, POMP, SLC27A4, SNAP29, SPINK5, ST14, STS, SUMF1, TGM1, TGM5, VPS33B*	15 *BAP1, BRCA2, CDK4, CDKN2A, CHEK2, MC1R, MITF, MUTYH, POT1, PTEN, RB1, SLC45A2, TERT, TP53, TYR*
Invitae	46* AAGAB, AQP5, ATP2C1, CAST, CD151, CDSN, COL17A1, COL7A1, CTSC, DSG1, DSP, DST, ENPP1, EXPH5, FERMT1, GJB6, ITGA3, ITGA6, ITGB4, JUP, KANK2, KLHL24, KRT1, KRT10, KRT14, KRT16, KRT17, KRT5, KRT6A, KRT6B, KRT6C, KRT9, LAMA3, LAMB3, LAMC2, LOR, PKP1, PLEC, POMP, RHBDF2, RSPO1, SERPINB7, SERPINB8, SLURP1, TAT, TRPV3	46 *ABCA12, ABHD5, ALDH3A2, ALOX12B, ALOXE3, AP1S1, AQP5, CAST, CDSN, CERS3, CLDN1, CYP4F22, EBP, ELOVL1, ELOVL4, GJA1, GJB2, GJB3, GJB4, GJB6, KDSR, KRT1, KRT10, KRT2, KRT9, LIPN, LOR, MBTPS2, NIPAL4, PEX7, PHYH, PNPLA1, POMP, SDR9C7, SERPINB7, SERPINB8, SLC27A4, SNAP29, SPINK5, ST14, STS, SULT2B1, SUMF1, TGM1, VPS33B, ZMPSTE24*	9 *BAP1, BRCA2, CDK4, CDKN2A, MITF, POT1, PTEN, RB1, TP53*
Blueprint	26 *ATP2C1, CDSN, COL17A1, COL7A1, CSTA, DSG1, DSG2, DSG4, DSP, DST, EXPH5, FERMT1, GRIP1, ITGA3, ITGA6, ITGB4, KLHL24, KRT1, KRT14, KRT5, LAMA3, LAMB3, LAMC2, PKP1, PLEC, TGM5*	39 *ABCA12, ABHD5, ALDH3A2, ALOX12B, ALOXE3, CASP14, CDSN, CERS3, CSTA, CYP4F22, EBP, ERCC2, FLG, GJA1, GJB2, GJB3, GJB4, KDSR, KRT1, KRT10, KRT2, KRT9, LIPN, LOR, MBTPS2, MPLKIP, NIPAL4, OSMR, PEX7, PHYH, PNPLA1, SDR9C7, SLC27A4, SPINK5, ST14, STS, SUMF1, TGM1, TGM5*	19 *BAP1, BRCA1, BRCA2, CDK4, CDKN2A, DDB2, ERCC2, ERCC3, ERCC4, ERCC5, MITF, POT1, PTCH1, PTEN, SUFU, TP53, WRN, XPA, XPC*
Prevention Genetics	18 *COL17A1, COL7A1, DSP, DST, FERMT1, ITGA3, ITGA6, ITGB4, JUP, KRT10, KRT14, KRT5, LAMA3, LAMB3, LAMC2, PKP1, PLEC, TGM5*	19 *ABCA12, ABHD5, ALOX12B, ALOXE3, AP1S1, CERS3, CLDN1, CYP4F22, KRT1, KRT10, KRT2, KRT9, LIPN, NIPAL4, PNPLA1, POMP, SLC27A4, ST14, TGM1*	10 *BAP1, BRCA2, CDK4, CDKN2A, CHEK2, MITF, POT1, PTEN, RB1, TP53*
CTGT	24 *CAST, CDSN, CHST8, COL17A1, COL7A1, CSTA, DSP, DST, EXPH5, FERMT1, ITGA3, ITGA6, ITGB4, JUP, KLHL24, KRT14, KRT5, LAMA3, LAMB3, LAMC2, PKP1, PLEC, SERPINB8, TGM5*	32 *ABCA12, ALOX12B, ALOXE3, CASP14, CAST, CDSN, CERS3, CHST8, CSTA, CYP4F22, FLG, FLG2, GJA1, GJB3, GJB4, KDSR, KRT1, KRT10, KRT2, KRT83, LIPN, LOR, MBTPS2, NIPAL4, PNPLA1, POMP, SERPINB8, ST14, STS, SULT2B1, TGM1, TGM5*	

*Combined with palmoplantar keratoderma. Numbers show size of gene panel.

### Other genetic aberrations in melanoma

Tumor mutational burden is defined as the number of non-synonymous mutations per million bases and correlates with the amount of neoantigens in tumors. Immunotherapy with checkpoint inhibitors is more effective in treating tumors with higher levels of this biomarker (110, 111). Early studies utilized WES, however this has been extended to targeted panels (111). NGS panel-based determination of TMB show high concordance between TMB predicted by NGS panels and that using WES (109). The commercial panel FoundationOne CDx reports TMB and has gained approval in 2020 as the companion diagnostic for pembrolizumab with high TMB (>10 mutations/Mb).

One class of driver mutations arise from gene fusions in tumors. Traditionally, gene fusions have been detected at the protein level by immunohistochemistry (IHC), or at the DNA level by fluorescence *in-situ* hybridization (FISH). Even though some DNA-based NGS assays have been developed for the intronic detection of gene fusions, RNA-based NGS has shown higher sensitivity for the detection of fusion transcripts, by sequencing fused exons from different genes (intergenic fusions) or exon skipping (intragenic fusions) (112). In advanced stage non-small cell lung cancer, testing for clinically relevant gene fusions such as those driven by the *ALK* or *ROS1* genes is recommended by national guidelines, since these fusions can be targeted by small molecule inhibitors, such as crizotinib (113). *ALK* fusions have been detected in Spitz nevi, Spitz tumors, and Spitzoid melanomas, allowing to further characterize these diagnostically challenging tumors (114). Beyond *ALK* and *ROS1*, although of rare occurrence, *NTRK1/2/3* rearranged tumors demonstrates remarkable responsiveness to larotrectinib and entrectnib in a tumor type-agnostic manner (115).

## Cutaneous lymphoma

Cutaneous lymphomas (CL) are a heterogeneous group of lymphomas that present in the skin. The two main types of CLs are cutaneous T-cell lymphomas (CTCL) and cutaneous B-cell lymphomas (116). CTCL is much more common than cutaneous B-cell lymphomas, with Mycosis Fungoides (MF) and Sezary Syndrome (SS) representing the most common subtypes of CTCL (117). This section of our review will discuss how NGS has advanced the understanding of CLs by improving its diagnostic sensitivity, therapy response monitoring and prognosis predictions, and identifying possible pathogenic mechanisms and inspiring potential targeted treatment options.

### Diagnosis, therapy monitoring, and prognosis predictions

A principal diagnostic test for MF is the T-cell receptor (TCR) clonality assay (118). Polymerase chain reaction (PCR) coupled with capillary electrophoresis (CE) is the most widely used method. However, this PCR-CE method often produces ambiguous results due to the low abundance of clonal T lymphocytes, which results in clonal peaks that are weak and cannot be size-resolved by CE. NGS, on the other hand, has been found to have increased specificity and sensitivity for T-cell clonality detection over previous techniques (119). For example, a study with 35 MF patients found that 85% were found to have a clonal T-cell rearrangement using NGS, compared to just 44% using CE–based detection (118). Additionally, NGS of TCR in MF and SS patients was found to provide increased specificity and sensitivity when compared to flow cytometry and PCR (120).

NGS has also allowed researchers to monitor the therapeutic response and minimal residual disease in CTCL patients, which can significantly improve patient management during the long period of remission that MF and SS patients often enter after bone marrow transplantation (120). Discoveries using NGS technologies have also enhanced prognosis predictions in CTCL. For example, Park et al. used WGS among other genomic analyzes and found PD1 deletions to sufficiently reverse the exhaustion phenotype of T-cells (observed in PD1 wild-type), enhance the proliferation of lymphoma cells, and result in diminished rates of survival (121). In this way, PD1 deletions may now be considered an indication of worse prognosis for CTCL.

### Identification of recurrent mutations and signaling pathways with roles in pathogenesis and targeted treatment

Using NGS, researchers have identified numerous genetic mutations in CTCL that have shed light on possible pathogenic mechanisms and potential options for targeted therapy (Table 5). Park et al. used WGS among other genomic analyzes to identify 86 putative driver genes for CTCL, 19 of which had not yet been implicated in CTCL (121). Targeted therapies against these recently identified driver genes may have the potential to improve clinical outcomes for CTCL patients. Another study used targeted sequencing to sequence 585 genes linked to cancer in 71 skin or blood samples from 61 CTCL patients (117). The study identified recurrent mutations in tumor suppressor genes (*TP53*, *FAT1*, *FAT3*), as well as in genes responsible for chromatin remodeling (*ARID1B*), methylation of DNA (*DNMT1*) and histone (*MLL2*, *MLL3*, *KDM6A*), DNA mismatch repair (*MSH3*) and DNA damage response (*ATM*, *MDC1*) (117). All of which may play a role in CTCL pathogenesis. Additionally, Jones et al. used NGS and found CTCL to express signature 7 (123) – a mutational signature that has characteristics of UV induced mutations and is commonly found in malignant skin cancers such as melanoma and squamous carcinoma (125). Signature 7 was found to contribute to 52 and 23% of the mutational burden in MF and SS, respectively (123). In fact, analysis of data from the British 100,000 WGS project found that CTCL cases were the only non-Hodgkin’s lymphoma cases to express Signature 7 (123). These findings suggest that UV radiation may play a role in the lymphomagenesis of CTCL. Furthermore, Chang et al. collected and re-analyzed genomic data of 139 patients with MF or SS from seven separate NGS studies and identified 125 genes to be significantly mutated (*p* < 0.05). Notably, *TP53* was one of the most commonly mutated oncogenes and was detected in 19% of cases (122). Furthermore, NGS can also be used to identify germline variants that may increase cancer risk. For example, Gross et al., used NGS to identify a germline *BRCA2* mutation in a pediatric patient with transformed MF. This signifies how NGS has the potential to identify at-risk family members, particularly in families with familial cancer syndromes and germline mutations, so that necessary cancer screening and other risk-reducing measures can be implemented (126).

**Table 5:** Mutations identified using NGS in CTCL.

Mutation category	Mutation	Reference
Tumor suppressor genes	*ARID1A, DNMT3A, MSH2, PDCD1, TMCC1, NR3C1, ATXN1, HLA-B/C, TNFA/P3, FOXO3, AHR, LATS1, EGR3, CDKN2A, HNPRNK, TGFBR1, ZEB1, AGAP6, FAS/PTEN, MGMT, WT1, ATM, CDKN1B, SOCS2, RB1, ZFPM1, TP53, GRAP, ZBTB7A, SBNO2, MAP4K1, PD1, FUBP1, ANO6, BACH2, NFKB2, CTCF, FAT1, FAT3*	(117, 121)
Oncogenes	*IRF4, CARD11, PTPRN2, JAK2/PD-L1/PD-L2, PRKCQ, TP53, PLCG1, FAS, POT1, DNMT3A, KIT, TNFRSF1B, RHOA*	(121, 122)
Hotspot point mutations	*NFKB1, KLF2, JUNB, TBL1XR1*	(121)
Enrichment of mutational signatures	Signature 1 (related to aging), Signature 7 (related to UV induced mutations), Signature 11 (related to alkylating agents), Signature 17 (possibly related to oxidative damage)	(121, 123)
Chromosome arm-level somatic copy number variants (SCNVs)	17p deletion, 10q deletion, 17q amplification in Leukemic CTCLs	(121)
Signaling pathways	• Receptor Tyrosine Kinase (*IRS2, FOXO3*) • JAK/STAT (*JAK3, STAT5A/B, SOCS1*) • NOTCH (*NOTCH1, NOTCH2*) • NF-kB pathway (*PLCG1, CARD11, TNFRSF1B, KIT*) • TP53 pathway • PI3K-serine/threonine protein kinases (*AKT*) • Fibroblast growth factor receptors (*FGFR*) • Peroxisome proliferator-activated receptors (*PPAR*) • T-cell–specific pathways (*MAP4K1, ANO6, GRAP, NR3C1, SBNO2, SOCS2, BACH2, NFKB1, KLF2, JUNB, AHR, ZFMP1, ZBTB7A*)	(117, 121, 122, 124)
DNA damage repair and epigenetic	*ATM, MDC1, MSH3, ARID1B, MLL2, MLL3, KDM6A, DNMT1*	(117)
Miscellaneous	Androgen Receptor (*AR*) (subclonal level)	(117)

NGS has also shed light on the numerous altered signaling pathways of CTCL. Chang et al. showed CTCL patients to have mutations in the nuclear factor-kappaB (NF-κB) pathway (122). Constitutive activation of this pathway has been found to be involved in apoptosis resistance in CTCL tumor cells, therefore targeting this pathway may have therapeutic effects (127). In fact, a phase II clinical trial showed Bortezomib, a NF-κB signaling inhibitor, to be well tolerated with an overall response rate of 67% in individuals with relapsed or refractory CTCL (128). In addition, Chang et al. found TP53 and NF-κB gene pathway mutations to be mutually exclusive, suggesting that tumor variants may originate from distinct genetic backgrounds (122). Furthermore, it was found that gene mutations within NF-kB pathway exhibited mutual exclusivity, which indicates that the pathogenesis of CTCL may be induced by only one pathway. Lastly, the researchers found that patients who did not have p53 or NF-κB pathway gene mutations also did not express any other significant mutations. This suggests that lymphomagenesis may be triggered by other significant alterations in the transcriptome or epigenome. Beyond the NF-κB pathway, NGS studies have identified other recurrently altered signaling pathways in CTCL patients which may play a role in CTCL pathogenesis, such as JAK–STAT, PI3K-serine/threonine protein kinases, fibroblast growth factor receptors, and peroxisome proliferator-activated receptors (124).

The use of NGS has allowed for the identification of diverse genetic mutations and has implicated numerous altered signaling pathways to be involved in CTCL pathogenesis. These new insights have the potential of guiding future targeted therapies to improve CTCL patient outcomes.

## Discussion and conclusions

NGS has offered distinct advantages to prior genetic testing techniques in applications where numerous genes require sequencing. Given the inherent heterogeneity of genodermatoses and cutaneous malignancies, NGS DNA sequencing offers an efficient means of testing across a large range of possible mutations. Advantages also exist in the ability for digital sequencing results to be quantitated allowing sensitive analysis of clonal cell populations, such as in CTCL.

The use of NGS for diagnosis of EB and ichthyosis exemplifies the potential applications of NGS for genodermatoses in general. The diagnostic yield in these two groups of genodermatoses has among the highest diagnostic yields among genetic diseases, attributable to the well-characterized mutational spectrum (28). The ability of NGS to sequence across the entire region of many genes in parallel and at great depth allows accurate diagnosis of clinically heterogeneous diseases. Due to the decreasing costs of sequencing and improved ease of workflows, NGS testing is increasingly being considered as the first-line testing for many genodermatoses.

For melanoma, NGS panels have become a powerful tool for molecular characterization. NGS is increasingly being used in the clinical diagnosis and management of melanomas. Melanomas show among the highest yield of actionable targets, attributable to prevalence of hotspot *BRAF* mutations and other well characterized mutations. Furthermore, TMB has further improved NGS’s role in management. NGS has shown superiority to traditional Sanger-based diagnostics particularly in the ability to sequence large number of genes in parallel. This is potentially beneficial in avoiding errors due to missed reflex testing of *NRAS* and *KIT* in *BRAF* WT melanomas (129). Compared to Sanger sequencing, NGS shows comparable cost, with one study showing slightly lower cost at €415 EUR per sample versus €465 with conventional testing (94). This is expected to decrease further with improvements in sequencing technology. As with timing, sequencing results are variable depending on the analytical demands on the backend. One study showed completion of NGS panels in three working days, shorter than conventional methods (94). With automated validated analysis, such as with PGDx’s platform, turnaround with commercial panels can be as fast as 4-5 days.

### Limitations

NGS has some limitations that need to be addressed before it can be widely adopted, including issues with speed, cost, technical limitations, and availability.

In the case of EB, NGS has not entirely supplanted biopsy-based IFM due to limitations including turnaround time. Compared to IFM, which can provide diagnosis within hours to days, NGS techniques, such as WGS and WES in practice takes weeks, while targeted sequencing may be performed more rapidly on the order of days. Current turnaround time for most commercial tests range from 2 to 4 weeks, precluding first-line use of NGS testing in early and severe cases where prompt management and assessment of prognosis is required. This is illustrated in practice by the more frequent use of IFM early in life with severe cases, while genetic testing is used later. The median age genetic analysis was 24.5 months compared to 1.0 month for IFM (19). Optimization of steps can reduce turnaround time as demonstrated in an EB cohort where authors describe a 72-h procedure as well as availability of rapid commercial WES tests with verbal results available in 7 days (27). One of the key bottlenecks regarding timing is analysis and identification of pathogenic variants. While analysis from published reports is often manually intensive, improvement in databases of known pathogenic variants and automation of analysis pipelines can shorten turnaround time.

In terms of increased turnaround time due to the sequencing step, throughput of sequencing platforms is important given the rarity of these conditions where very few samples will be sequenced at a time. Many of the studies reviewed utilize low throughput devices such as the MiSeq (Illumina) and Ion Torrent (Thermo Fisher). Newer small-scale platforms are being produced that are ideal for targeted sequencing panels. Alternatively, sequencing individual gene panels or exomes can be run together with other applications on high-throughput platform, allowing decreased costs of testing.

Another often cited limitation of NGS testing is cost. While testing costs on the order of hundreds to one thousand United States dollars, these costs are similar if not lower than conventional testing methods. In early estimates of cost, authors in 2012 commented that the cost of WES was similar to skin biopsy analysis with IFM and TEM (130). Other authors noted that the cost of WES was similar to sequencing the *COL7A1* gene locus alone at approximately £900 GBP (16), and that a targeted sequencing panel was estimated to cost even less at €350 EUR (27). Recent cost analysis of targeted sequencing of EB in Brazil noted that while higher in cost compared to IFM (R$ 800 vs. R$ 500 BRL), the greater diagnostic efficiency supported use as first-line diagnostic (131).

There are also technical limitations of NGS in genome coverage. In many studies, large copy number variations were noted to be missed with NGS techniques. In an early study, a patient with previously characterized whole-exon deletion was not detected by NGS (36). One challenge with NGS in melanoma is the choice between whole exome versus targeted panels. While targeted panels are currently optimized for cancer-related genes, detection of structural variants beyond well-established single nucleotide variants is challenging. Structural variants are much more prevalent in mucosal and acral melanomas as shown through whole genome sequencing studies (86, 132). Due to limitations in the detection of genomic events driving melanomas in these locations, techniques with broader coverage are needed. Newer analysis tools with improved performance in calling copy number variations have become available that may allow improved identification.

Finally, availability of testing has been another limitation to NGS testing. The infrastructure needed for NGS testing requires not only sequencing capabilities, but also bioinformatic support. While most studies referred to in this review utilize in-house custom sequencing panels and analysis pipelines, commercial versions of EB, ichthyosis, and several other disease-focused gene panels are available and have been used in published studies (42). Availability of NGS testing for EB is available in at least six commercial labs (GeneDx, Fulgent, Invitae, Blueprint Genetics, Prevention Genetics, CTGT) and several university labs (Table 4). Tests for diseases are listed and searchable at the NIH Genetic Testing Registry.

Wider adoption has been limited by cost and timing of this technique. With improved scale and technological improvements in sequencing platforms, cost has been decreasing, meeting parity with traditional techniques for many applications. Similarly, with improved characterization of the spectrum of genetic mutations as well as improved algorithms for identifying mutations, the timing will also continue to shorten. In practice, know-how of NGS technology is not necessary as many labs and commercial services offer many tests. Among well characterized genodermatoses, such as EB and ichthyosis, specific panels have been curated to allow for high sensitivity testing. Similarly, for malignancies, cancer gene panels allow testing for the most common genetic mutations.

The possible applications of NGS also go well beyond that covered within the scope of this review. Novel applications not covered within the scope of this review also further underscore the utility of NGS, including pre-implantation genetic testing, liquid biopsies testing cell-free tumor DNA in patient serum, as well as whole exome sequencing for neoantigen identification for use in personalized immunotherapy. Beyond DNA sequencing, other techniques exist including sequencing of RNA, which has many applications including improving detection of variants in genodermatoses and malignancies. Other techniques such as DNA methylation, chromatin modification, chromatin accessibility, and single cell sequencing are used frequently in research and have potential applications in clinical Dermatology.

## Author contributions

AK, HD, and PK contributed to writing. AK, HD, and DM contributed in conception. All authors contributed to the article and approved the submitted version.

## Conflict of interest

The authors declare that the research was conducted in the absence of any commercial or financial relationships that could be construed as a potential conflict of interest.

## Publisher’s note

All claims expressed in this article are solely those of the authors and do not necessarily represent those of their affiliated organizations, or those of the publisher, the editors and the reviewers. Any product that may be evaluated in this article, or claim that may be made by its manufacturer, is not guaranteed or endorsed by the publisher.

## Abbreviations

NGS: Next-generation sequencing
WGS: Whole-genome sequencing
WES: Whole-exome sequencing
EB: Epidermolysis bullosa
IFM: Immunofluorescence antigen mapping
TEM: Transmission electron microscopy
ARCI: Autosomal recessive congenital ichthyosis
KPI: Keratinopathic ichthyosis
PPKs: Palmoplantar keratodermas
cSCC: Cutaneous Squamous Cell Carcinoma
UV: Ultraviolet
CSD: Cumulative solar damage
TMB: Tumor mutational burden
TCR: T-cell receptor
IHC: Immunohistochemistry
FISH: Fluorescence *in-situ* hybridization
CL: Cutaneous lymphoma
CTCL: Cutaneous T-cell lymphomas
MF: Mycosis Fungoides
SS: Sezary Syndrome
CE: Capillary electrophoresis
